# Characterizing Mobility and its Association with HIV Outcomes in Refugee Settlements in Uganda

**DOI:** 10.5334/aogh.4367

**Published:** 2024-03-25

**Authors:** Robin E. Klabbers, Canada Parrish, Patient Iraguha, Marcel Kambale Ntuyenabo, Scovia Ajidiru, Valentine Nshimiyimana, Kampire Caroline, Zikama Faustin, Elinor M. Sveum, Timothy R. Muwonge, Kelli N. O’Laughlin

**Affiliations:** 1 Department of Emergency Medicine, University of Washington, Seattle, WA, United States of America; 2 Department of Global Health, University of Washington, Seattle, WA, United States of America; 3 Infectious Diseases Institute, Makerere University, Kampala, Uganda

**Keywords:** Refugee, Uganda, mobility, sub-Saharan Africa, HIV, retention

## Abstract

**Background:**

A better understanding of refugee mobility is needed to optimize HIV care in refugee settlements.

**Objectives:**

We aimed to characterize mobility patterns among people living with HIV in refugee settlements in Uganda and evaluate the association between mobility and retention in HIV care.

**Methods:**

Refugees and Ugandan nationals accessing HIV services at seven health centers in refugee settlements across Uganda, with access to a phone, were recruited and followed for six months. Participants received an intake survey and monthly phone surveys on mobility and HIV. Clinic visit and viral suppression data were extracted from clinic registers. Mobility and HIV data were presented descriptively, and an alluvial plot was generated characterizing mobility for participants’ most recent trip. Bivariate Poisson regression models were used to describe the associations between long-term mobility (≥1 continuous month away in the past year) and demographic characteristics, retention (≥1 clinic visit/6 months) and long-term mobility, and retention and general mobility (during any follow-up month: ≥2 trips, travel outside the district or further, or spending >1–2 weeks (8–14 nights) away).

**Findings:**

Mobility data were provided by 479 participants. At baseline, 67 participants (14%) were considered long-term mobile. Male sex was associated with an increased probability of long-term mobility (RR 2.02; 95%CI: 1.30–3.14, p < 0.01). In follow-up, 185 participants (60% of respondents) were considered generally mobile. Reasons for travel included obtaining food or supporting farming activities (45% of trips) and work or trade (33% of trips). Retention in HIV care was found for 417 (87%) participants. Long-term mobility was associated with a 14% (RR 0.86; 95%CI: 0.75–0.98) lower likelihood of retention (p = 0.03).

**Conclusions:**

Refugees and Ugandan nationals accessing HIV care in refugee settlements frequently travel to support their survival needs. Mobility is associated with inferior retention and should be considered in interventions to optimize HIV care.

## Introduction

Refugees are frequently characterized as a mobile population [1]. The term ‘refugee’ itself implies movement: fleeing from conflict or persecution in one country and crossing an international border to find safety in another [2]. Much of the research that has been conducted on refugee mobility has focused on mobility in transit or mobility after permanent or semi-permanent relocation in the Global North [3,4,5]. Less is known about the mobility patterns of refugees after they have been granted asylum in the Global South.

Sub-Saharan Africa is home to 7.1 million refugees, or 20% of the global refugee population [6]. In sub-Saharan Africa, mobility is closely linked to income generation and constitutes an essential livelihood strategy. For refugee populations who often face food scarcity, have limited livelihood options available to them, and are more vulnerable to economic shocks, the economic drivers of mobility are likely amplified. Social drivers of mobility may also be heightened for refugees as a result of disrupted social networks and separated families. The close proximity of host countries to countries of origin in sub-Saharan Africa, coupled with the ease of movement across borders, may facilitate refugee mobility between countries [7].

Refugee mobility is important in the context of HIV. Research among non-refugee populations demonstrates that mobility is associated with lower uptake of HIV testing [8], an increased likelihood of loss to follow-up or disengagement from care [9,10,11,12], a higher probability of antiretroviral therapy (ART) non-adherence [13,14], and lower rates of HIV viral suppression [15,16]. A recent publication by Thorp et al. highlighted the need to unpack the concept of mobility and gain a better understanding of the types of mobility that most impact HIV outcomes, as well as the demographic profiles associated with high-risk mobility [17]. As conflict and violence continue to rise globally and the impacts of climate change worsen, forced migration will become more prevalent. Against this backdrop, understanding how mobility impacts HIV outcomes will be vital to the optimization of HIV programs in refugee settlements.

In this study, we aimed to characterize the mobility patterns of refugees and Ugandan nationals living with HIV accessing HIV services in refugee settlements in Uganda and to evaluate the association between mobility and retention in HIV care.

## Methods

### Study setting

As of July 2023, Uganda was home to approximately 1.6 million refugees [18]. In Uganda, the vast majority of refugees (95%) live in refugee settlements located in the border regions in the southwest, midwest, and northern parts of the country. In southwestern Uganda, refugees mainly come from the neighboring Democratic Republic of the Congo, Burundi, and Rwanda, while in northern Uganda, most refugees come from South Sudan. Borders between Uganda and neighboring countries are porous, and refugees travel freely between countries.

When refugees come to Uganda, they are given a small plot of land in a refugee settlement to cultivate [19]. Refugees are free to settle anywhere in the country, but humanitarian assistance is restricted to the refugee settlements. Livelihood opportunities in refugee settlements are limited, and many refugees rely on subsistence farming supplemented with food distributions or cash transfers provided by humanitarian organizations to meet their survival needs. While intended only for refugees, the refugee settlements are also home to a minority of Ugandan nationals.

In refugee settlements, HIV services, including HIV testing and ART, are freely available for refugees and Ugandan nationals. In 2021, HIV prevalence among refugees >15 years old living in refugee settlements in Uganda was 1.5% [20]. Refugees face barriers to HIV care engagement, and only 74% of individuals newly diagnosed with HIV in the Nakivale Refugee Settlement between 2018 and 2020 were linked to HIV care [21]. The current study was conducted at 7 health centers offering HIV testing and treatment; these health centers were located in Nakivale Refugee Settlement (southwest; Nakivale HC III, Juru HC II, Kibengo HC II), Adjumani Refugee Settlement (north; Pagrinya HC III, Ayilo HC III), and Palorinya Refugee Settlement (north; Itula HC, Palorinya HC III).

### Study Design and implementation

#### Participants

Refugees and Ugandan nationals ≥18 years of age, living with HIV (either newly testing positive for HIV or previously enrolled in HIV care), accessing HIV services at participating health centers, with access to a phone were eligible to participate. Eligible participants were recruited at the time of HIV testing, during routine HIV follow-up appointments, or by phone using contact information listed in clinic HIV registers.

#### Data collection

Eligible individuals interested in participating were read a consent form in their language of choice (Kiswahili, Kinyarwanda, Runyankore, Somali, Arabic, Kakwa, Dinka, Lugbara, or English) by a research assistant with the help of an interpreter when necessary. Following written informed consent, contact information, socio-demographic data (sex, age, refugee status, relationship status, education, time living in settlement), and mobility history (travel in the past year, reasons for travel, relocation frequency) were collected in an intake survey administered verbally by the research assistant, with answers entered directly into a mobile REDCap database with offline capabilities. Following the intake survey, all participants who had recently tested positive for HIV were linked to the HIV clinic to initiate ART per standard protocol.

**Figure 1:**
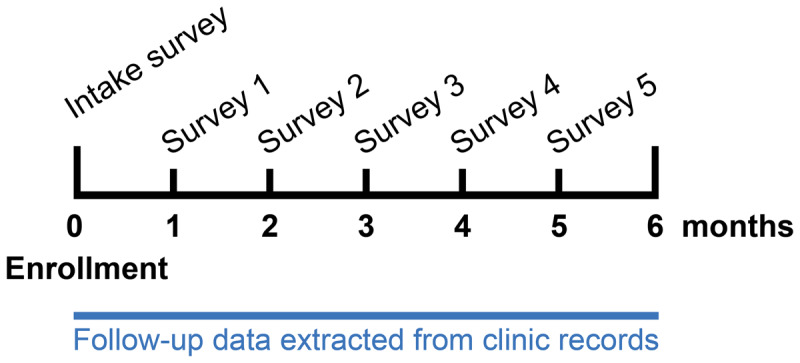
Data collection timeline.

Starting one month after enrollment, each month for five months, participants received a pre-recorded interactive voice response (IVR) phone survey in their study language of choice (Figure 1). Participants were able to respond to multiple-choice survey questions by pressing numbers on their telephone keypad. They were asked questions about mobility in the past four weeks (10 questions) and questions about HIV perceptions and their HIV care (10 questions) (complete survey in Appendix 1). Various aspects of mobility were captured in the survey, including change in place of residence, number of nights spent away from home, distance of travel (outside country > district > subcounty > parish > village), frequency of travel, and details regarding their most recent trip. Participants indicating they did not travel in the last month did not receive any subsequent survey questions. To prevent unintentional HIV disclosure to individuals other than the participant in the call, participants were asked to enter the three-digit study passcode that was given to them at enrollment before gaining access to the second half of the survey, which contained HIV-related content. Participants were compensated UGX 20,000 (~$5.49, €4.94) in mobile money, which was transferred to their phone upon completion of the survey.

Participants were followed-up for six months from enrollment. For this follow-up period, all available data on clinic attendance and viral load were extracted from participants’ HIV clinic records.

### Exposures

The exposure of interest in this study was participant mobility (Table 1). Long-term mobility was considered a dichotomous variable and was determined based on baseline characteristics collected in the intake survey. Long-term mobility was defined as spending ≥1 continuous month away from home in the past year. In a sensitivity analysis, an alternative definition of mobility was explored based on participants’ answers to prospectively collected monthly mobility surveys. In this alternative definition, participants were deemed generally mobile if, during any of the monthly surveys, they reported taking ≥2 trips, traveling outside the district or further, and/or spending >1 week to 2 weeks (8–14 nights) away from home, i.e., participants were deemed generally mobile if travel was frequent, far, or long.

**Table 1:** Mobility definitions.


	DEFINITION	CHARACTERISTICS CAPTURED	SOURCE

Long-term mobility	Spending ≥1 continuous month away from home in the past year	Duration	Intake survey at enrollment

General mobility	Any of the following during any follow-up month: Taking ≥2 trips Traveling outside the district or further Spending >1 week to 2 weeks (8–14 nights) away from home	Frequency Distance Duration	Monthly phone survey (answers aggregated across 5 survey time points)


### Outcomes

The primary study outcome was retention in HIV care, a dichotomous outcome (retained, yes/no) in which a participant was considered retained if they had ≥1 HIV clinic visit recorded in their HIV register within the 6-month study follow-up period, excluding the day of enrollment. This definition of retention was based on local HIV clinical guidelines at the time the study was conducted, which allowed for 6-month (instead of 3-month) ART dispensing for stable clients in response to the COVID-19 pandemic [22].

Secondary outcomes included linkage to HIV care and viral suppression. A participant was considered linked to HIV care (dichotomous outcome, yes/no) if a newly diagnosed index client (diagnosed with HIV in the past 90 days) had ≥1 HIV clinic visit recorded in their HIV register within 90 days of diagnosis, excluding the day of testing. Participants were considered virally suppressed (dichotomous outcome, yes/no) if they had a viral load measurement <1,000 copies/mL recorded in the HIV register in the 6-month study follow-up period [23].

### Analysis

Simple frequency counts and proportions were reported for baseline demographic characteristics, baseline mobility, prospectively collected monthly mobility data, HIV-related survey findings, and study outcomes. Monthly mobility data were stratified and presented by sex, food security status, and refugee/Ugandan national status. An alluvial plot, a type of flow diagram used to visualize streams of data across categorical variables, was generated using participant survey data on their most recent trip that month, with the answers from the five survey timepoints aggregated in a complete case analysis. Bivariate Poisson regression models with log links and robust standard errors were used to estimate the relative probability of being retained in HIV care, comparing mobile participants to non-mobile participants. Models were fit for long-term and general mobility. Additionally, bivariate Poisson regression models with log links and robust standard errors were fit to examine the association between various demographic characteristics (sex, education, age, relationship status, refugee status, place of residence, language) and baseline mobility to identify characteristics predictive of mobility.

### Ethical considerations

Ethical approval to conduct this study was obtained from the Makerere University School of Health Sciences Research Ethics Committee (MAKSHSREC: 2020–36, 11/16/2020) and the University of Washington Human Subjects Division (STUDY00011181, 10/13/2020). In addition, in accordance with national guidelines in Uganda, clearance to conduct this study was also obtained from the Uganda National Council for Science and Technology (HS1197ES). Informed consent was obtained from all participants prior to study participation.

## Results

Between September 6, 2021, and April 1, 2022, 629 participants (31 newly diagnosed index clients and 598 index clients diagnosed >90 days prior) were recruited to participate in the study. Intake survey data and at least one completed round of the monthly mobility survey were available for 479 participants (24 newly diagnosed index clients and 455 index clients diagnosed >90 days prior) who were included in the analysis. Participants answered an average of 3.3 out of five possible monthly mobility surveys, providing mobility follow-up data for a total of 1,565 person-months. Question completion rates were higher for questions posed earlier in the survey than those posed later in the survey (Appendix 2). Most participants were female (68%) and had a mean age of 41 years (Table 2). Participants included 273 refugees and asylum seekers (57%) and 206 Ugandan nationals (43%). The most common countries of origin among refugee and asylum seeker participants were Rwanda, Somalia, and Burundi. One in four participants reported experiencing food insecurity.

**Table 2:** Participant characteristics.


	PARTICIPANT TOTAL (N = 479)

**DEMOGRAPHIC CHARACTERISTICS** ^1^	

Female, N (%)	326 (68%)

Age in years, mean (SD)	41 (12)

Relationship status, N (%)	

Married and cohabiting	239 (50%)

Married and living apart	33 (7%)

Cohabiting but not married	94 (20%)

Living apart and not married	97 (20%)

Divorced/separated	14 (3%)

Highest level of education, N (%)	

Never attended school	342 (71%)

Some primary school	113 (24%)

Completed primary school	11 (2%)

Some secondary school	12 (3%)

Food insecure,^2^ N (%)	123 (26%)

Refugee status, N (%)	

Refugee	269 (56%)

Asylum seeker	4 (1%)

Ugandan national not internally displaced	186 (39%)

Ugandan national internally displaced	20 (4%)

Country of origin, N (%)	

Uganda	205 (43%)

Rwanda	98 (21%)

Democratic Republic of the Congo (DRC)	69 (14%)

Burundi	61 (13%)

South Sudan	35 (7%)

Sudan	5 (1%)

Kenya	3 (1%)

Ethiopia	2 (0.4%)

Tanzania	1 (0.2%)

Place of residence, N (%)	

Nakivale refugee settlement	279 (58%)

Palorinya refugee settlement	23 (5%)

Adjumani refugee settlement	16 (3%)

Outside of the settlement	154 (32%)

Years lived in refugee settlement, Median (IQR)	7 (5–12)

Years lived in the district (for those living outside the refugee settlement), Median (IQR)	20 (9–33)

Travel time to clinic in minutes (one-way), median (IQR)	60 (30–120)

**HIV CHARACTERISTICS**	

Newly diagnosed (diagnosed in past 90 days), N (%)	24 (5%)

Diagnosed >90 days prior, N (%)	455 (95%)


^1^ Percentage of total number of participants reported (N = 479). For each demographic characteristic, percentages, including missingness (not reported in this table), total 100%. ^2^ Participants were considered food insecure if at any time in the past 4 weeks there was no food to eat of any kind in their household because of a lack of resources, or if their household went to sleep at night hungry because there was not enough food at least 3–10 times.

### Baseline mobility

Participants commonly reported having lived at their current refugee settlement or district (for those living outside of a refugee settlement) for multiple years (median seven years for refugees, median 20 years for Ugandan nationals). At baseline, 67 participants (14%) were considered long-term mobile, having spent ≥1 continuous month away from home in the past year (Table 3). The most important reasons participants provided for being away were employment or trade (58%), being with nuclear or extended family and friends (19%), and farming or looking for food (13%). Of the demographic characteristics, in a logistic regression model, male sex (compared to female sex) was associated with an increased probability of baseline mobility (RR 2.02; 95% CI: 1.30–3.14, p = 0.002). Compared to participants in the youngest age category (18–24 years), participants in the oldest age category (55 years and older) were less likely to be mobile at baseline (RR 0.26; 95%CI: 0.08–0.85, p = 0.03).

**Table 3:** Mobility at baseline.


	PARTICIPANT TOTAL (N = 479)

Number of times home changed in the past 10 years, N (%)	

Once	138 (29%)

Twice	51 (11%)

Three times	15 (3%)

Four times	13 (3%)

Five or more times	6 (1%)

No home change	220 (46%)

Home change concerned move to a different country, N (% of those who reported a home change)	79 (35%)

Long-term mobility: spent ≥1 month away from home in the past year, N (%)	67 (14%)

Reasons for spending ≥1 month away, N (% of those who spent ≥1 month away)	

Employment or trade	39 (58%)

To be with partner or children, or visiting extended family or friends	13 (19%)

Farming or looking for food	9 (13%)

Education or studies	2 (3%)

Touring	2 (3%)

Attending a function (e.g. funeral, wedding)	1 (2%)

Other	1 (2%)


^1^ Percentage of total number of participants reported (N = 479). For each demographic characteristic, percentages, including missingness (not reported in this table), total 100%.

### Prospectively collected mobility data

The proportions presented are of person-months for which data on each topic was available (Appendix 2). Participants reported a residence change in almost a third of the follow-up timepoints (473/1,552 person-months, 30%); similar trends were observed for spending nights away from home (465/1,482 person-months, 31%). Travel often concerned only one trip (246/513 person-months, 48%) and lasted a short period of time; in 50% of cases (264/530 person-months), total monthly travel lasted only 1–2 nights, though in 5% of cases (24/530 person-months), travel was >3 weeks. Based on the monthly mobility surveys, 60% of participants (185/307 participants) could be considered mobile having taken ≥2 trips during any follow-up month, having traveled outside the district or further during any follow-up month, or having spent >1 week to 2 weeks away from home during any follow-up month (Table 4). Some differences in mobility could be observed by sex, food security status, and refugee/Ugandan national status (Appendix 3). Namely, female sex was associated with a higher prevalence of home change in the past 4 weeks (p < 0.01), spending nights away from home was more prevalent among participants who were food insecure (p = 0.03), and refugees were more likely than Ugandan nationals to report a home change or having spent nights away from home in the past 4 weeks (p < 0.001 and < 0.01, respectively).

**Table 4:** Prospectively collected mobility.


	PARTICIPANT TOTAL (N = 307)^1^

Mobile (meet any of the criteria below), N (%)	185 (60%)

Frequency: took ≥2 trips during ANY month, N (%)	150 (49%)

Distance: travelled outside the district or further during ANY month, N (%)	68 (22%)

Duration: Spent >1 week to 2 weeks (8–14 nights) away in ANY month, N (%)	69 (23%)


^1^ Of the 479 study participants, 307 (64%) answered monthly mobility survey questions about travel frequency, distance, or duration.

**Figure 2:**
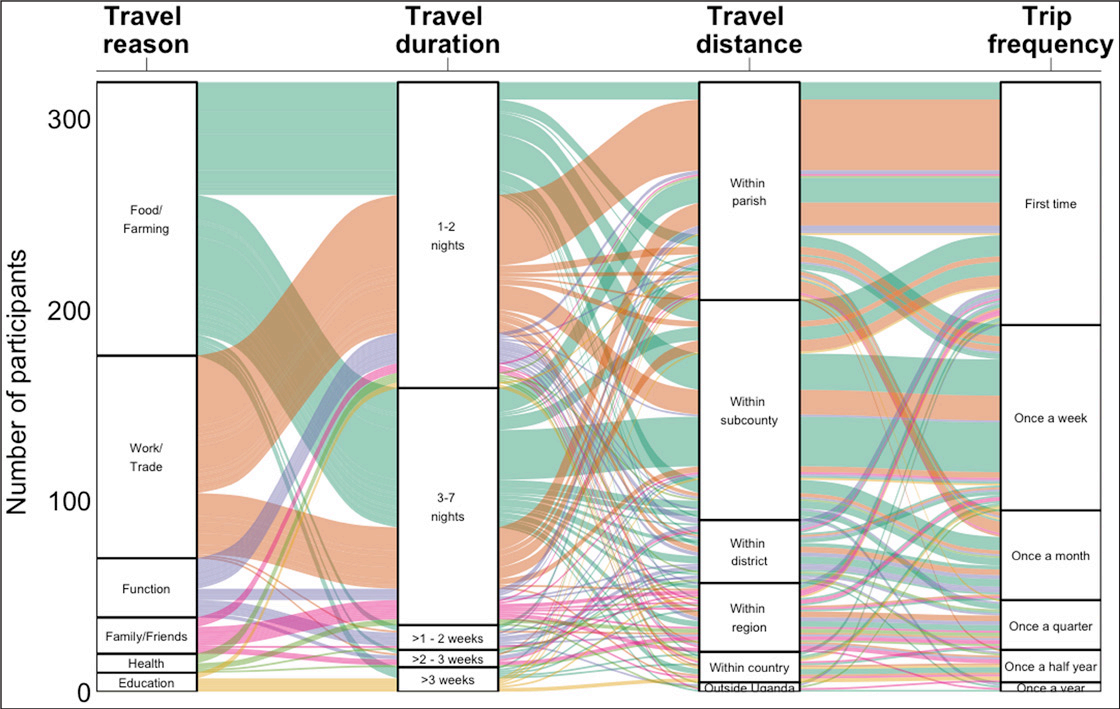
Alluvial plot showing the relationship between various travel characteristics for 319 recent trips reported by participants (N = 273). * All travel characteristics are mutually exclusive.

To gain a better understanding of mobility characteristics, participants were asked to answer questions specifically about their most recent trip. Participants provided information on their most recent trip for a total of 319 person-months. Proportions are presented of person-months for which data on each topic were available (Appendix 2). Most of these trips (143/319 person-months, 45%) were conducted to obtain food or to support farming activities (Figure 2). Trips to obtain food or support farming activities were typically short, lasting 1–2 nights (59/143 person-months, 41%) or 3–7 nights (73/143 person-months, 51%) and were mostly carried out within the subcounty (79/143 person-months, 55%) or even within the parish (34/143 person-months, 24%). These trips often concerned regular trips that were carried out weekly (62/143 person-months, 43%). In a minority of cases (6/143 person-months, 4%), however, travel to secure food lasted >3 weeks. Work and trade was the second most common reason for travel (106/319 person-months, 33%) and had a similar duration of 1–2 nights (72/106 person-months, 68%) or 3–7 nights (32/106 person-months, 30%). These trips were usually conducted closer to home within the parish (66/106 person-months, 62%), and often concerned first-time trips (59/106 person-months, 56%). Travel conducted for education (10/319 person-months, 3%) was often of a longer duration, with the majority of trips conducted for this purpose (7/10 person-months, 70%) lasting >3 weeks. Other reasons for travel included attending a function such as a wedding or a funeral (31/319 person-months, 10%), visiting family and friends (19/319 person-months, 6%), and health reasons (10/319 person-months, 3%). Travel outside of Uganda was less common (5/319 person-months, 2%) and was reported for a variety of reasons, including health, visiting family and friends, attending a function, and looking for food. One participant reported traveling outside of Uganda weekly for health reasons.

### Mobility and HIV care

Ninety-six unique participants (96/479 participants, 20%) provided answers to questions about mobility and HIV for 127 person-months. Proportions are presented of person-months for which data on each topic were available (Appendix 2). Respondents reported being on ART during almost all available follow-up timepoints (113/126 person-months, 90%). Over half of the time, respondents reported taking ART some of the time while on their most recent trip (57/110, 52%) and never taking ART during their trip for over a quarter of the time (29/110, 26%). The most important reason provided for not taking ART was not bringing enough medication (31/94 person-months, 33%), despite respondents reporting they received more ART than usual from their HIV clinic in preparation for their trip for the majority of person-months (86/111 person-months, 77%). Other important reasons for not taking ART included losing ART medication or having it stolen (20/94 person-months, 21%), forgetting to take ART (11/94 person-months, 12%), and being afraid others would discover their HIV status (13/94 person-months, 14%), as it was common for participants’ travel companions or the people at their travel destination to be unaware of respondents’ HIV status (58/127 person-months, 46% and 46/127 person-months, 36%, respectively). Despite these challenges, many respondents reported missing fewer ART doses while traveling (58/127 person-months, 45%), compared to when they are at home. As a result of their travels, almost half of respondents missed an HIV clinic appointment (56/116 person-months, 48%). Before their departure, respondents communicated with the HIV clinic about their trip for over a third of person-months (45/117 person-months, 38%) and sought care for HIV at a different health center while they were away for over a third of person-months (44/115 person-months, 38%).

### HIV care outcomes and their association with mobility

Of the 479 study participants, 417 participants (87%) had at least one clinic visit during the 6 month follow-up period and could be considered retained in care (Table 5). Of the newly diagnosed participants, 17 (71%) were linked to care and had at least one clinic visit within 90 days of diagnosis, excluding the day of testing. There was substantial missingness in viral suppression data, with no test data available in the 6-month follow-up window for 303 participants (63%). Among those for whom test data was available, a high prevalence of viral suppression was found (173/176, 98%). In bivariate logistic regression analysis, long-term mobility was associated with a 14% (RR 0.86; 95%CI: 0.75–0.98) lower likelihood of being retained in care. In a sensitivity analysis, “general mobility,” based on prospectively collected mobility data, was associated with a 3.0% (RR 0.97; 95%CI: 0.89–1.06) lower likelihood of being retained in care, though this result did not reach statistical significance. Given the high degree of missingness combined with the low prevalence of viremia, inferential analysis of the association between mobility and viral suppression was not feasible.

**Table 5:** HIV care outcomes.


	PARTICIPANT TOTAL (N = 479)

Retention in HIV care (≥1 clinic visit/6 month follow-up), N (%)	

Retained	417 (87%)

Not retained	62 (13%)

HIV care linkage (≥1 clinic visit in 90 days following diagnosis), N (%) for newly diagnosed index clients	

Linked	17 (71%)

Did not link to care	7 (29%)

Viral suppression (viral load < 1,000 copies/mL)	

Suppressed	173 (36%)

Non-suppressed	3 (0.6%)

Missing	303 (63%)


## Discussion

Refugees and Ugandan nationals living with HIV and accessing HIV care in refugee settlements in Uganda are a mobile population. Roughly 14% of participants reported spending a continuous month or more away from their home in the past year, and 60% of participants conducted long, far, or frequent trips during the six-month study period. Men were more likely to be mobile than women, and younger participants were more likely to travel than older participants. Travel was commonly conducted to secure basic survival needs such as food and income; it typically spanned a few days to a week, and 72% of trips did not reach beyond the subcounty. A minority of participants reported travel lasting multiple weeks and travel outside of the region. The responses of a subset of participants who answered HIV-related questions suggest that ART may not be taken consistently during travel due to logistical challenges and fear of HIV-related stigma, and that HIV clinic attendance may be negatively impacted by travel. While many participants reported never or only sometimes taking ART when they were traveling, notably, a subset of participants reported better adherence to ART during travel. In bivariate regression analysis, spending a prolonged period of time away from home was associated with a lower likelihood of retention in HIV care.

Our findings on refugee mobility are consistent with what has previously been described in the literature. Others have similarly found that mobility among refugees is strongly linked to obtaining the income and food necessary for daily survival. Ethnographic fieldwork conducted in Guinea revealed that Sierra Leonean refugees established business relationships, set up local and long-distance trade, and maintained crucial social ties through multi-directional cross-border movement [24]. In Kenya, Somali refugees commuted between refugee settlements and cities to buy and sell produce [25]. Among Liberian refugees in Ghana, three types of economic mobility were observed: movement between the refugee settlement and the capital Accra to sell and purchase goods and pursue employment opportunities, less frequent travel between the refugee settlement and Liberia for cross-border trade, and mobility between the settlement and subregional countries for trade in particular items [26]. Similar to our study findings, food has previously been identified as an important travel motivator among South Sudanese refugees in northern Uganda [27]. Lack of dependable food provision and cashflow sources in the refugee settlement led South Sudanese refugees to engage in cultivation across the border, where farming was said to be cheaper and easier, and to seize money-generating opportunities throughout Uganda when they were presented to them. In contrast to our study findings, the travel observed for South Sudanese refugees in Uganda was often for extended periods of time. Reasons for travel included visiting family and friends in other refugee settlements, undertaking secondary education at boarding schools (mainly boys), health, and business reasons.

In our study, we found that men were more likely to spend prolonged periods of time away from the refugee settlement than women. Differences in mobility profiles by sex have also been described by others [17,28,29]. Apopulation-based study in Kenya and rural Uganda (non-refugee populations) showed that men were more likely to travel for labor reasons, while women more often traveled for other reasons such as care-giving or -seeking, visiting family or friends, and attending a funeral [28]. Differences in mobility profiles by socioeconomic class have also been described. For Liberian refugees in Ghana, it was suggested that the majority of refugees participating in cross-border movement for economic reasons were better off and had sufficient assets to utilize mobility to their financial benefit [26]. Among South Sudanese refugees in Uganda, those conducting international business frequently had dependable access to resources (transport and trade goods) to support this travel [27]. Cross-border movement, however, was seen at both ends of the socio-economic spectrum, with the majority of people moving out of desperation.

To our knowledge, no studies have been published on the association between mobility and HIV care outcomes for refugees living in refugee settlements in sub-Saharan Africa. Outside the refugee settlement context, mobility has been associated with heightened HIV acquisition risk and inferior treatment engagement, though the heterogeneity of mobility measures and study designs has precluded synthesizing findings in a meta-analysis [30]. Our findings indicate a possible negative association between mobility and HIV care outcomes that is consistent with other populations.

Given the systemic drivers of mobility for refugee populations, it is unlikely that these behavioral patterns will change soon. To improve HIV care for refugee populations, it is imperative that mobility be considered when designing interventions to optimize HIV care outcomes. The UNAIDS Gap Report published in 2014 already made the case for developing migration-aware responses to HIV [31,32]. Ideally, interventions would ensure uninterrupted care access for refugees during travel, both inside and outside of host-countries. Potential solutions include the development of cross-border initiatives such as the cross-border forums on HIV and TB organized by the Musina Municipality in South Africa and their counterparts in neighboring Beitbridge, Zimbabwe [33]. Collaboration and communication across health centers within host countries are equally important. Biomedically-linked electronic medical records may play a facilitating role in fostering communication by making patient histories and treatment plans available to care providers everywhere. Though not ubiquitous, a 2022 report showed that mobile phones are owned by 81% of refugees in Nakivale, Bidi Bidi, Palorinya refugee settlement, and Kampala, suggesting that there may also be a role for mobile health (mHealth) interventions, for example through regular mobile check-ins between patients and providers while they are away and messaging to promote ART adherence [34]. Improving ART access for traveling refugees is also of importance, as evidenced in our study, in which participants reported bringing insufficient ART for their travels, losing ART medication, and having ART stolen during their trip. Currently, accessing services at other facilities may be challenging for mobile populations, as others have reported that official transfer letters are sometimes required in order to do so [17]. Finally, a proportion of refugees deemed lost to follow-up (LTFU) may have transferred out to other facilities [35]. Strengthening handoff and linkage of health records will be vital to correctly estimating LFTU rates and ART engagement for mobile populations.

The findings of this study should be considered in light of a number of study limitations. First, heterogeneity exists in the way mobility has been defined in the literature. In the absence of a unified understanding of this complex multi-dimensional phenomenon, we used a definition related to travel duration and, in sensitivity analysis, a composite measure of mobility that incorporated duration, distance, and frequency of travel. Study conclusions are dependent on how the exposure was defined, and different associations may have been found for alternative definitions of mobility. Second, one of the enrollment criteria for this study was access to a phone. The population with access to a phone in refugee settlements likely represents a group with higher socioeconomic status, limiting the external generalizability of study findings to the general refugee population. Third, while we initially set out to assess mobility longitudinally, high attrition and non-response among participants necessitated the aggregation of monthly surveys and conducting a cross-sectional assessment, hindering our ability to assess temporal associations between mobility and our study outcomes. Fourth, in addition to retention in HIV care, we also hoped to examine the association between mobility and viral suppression. Unfortunately, due to high missingness and only three participants with viral non-suppression in the dataset, there was insufficient data to ascertain this relationship. Fifth, due to the nature of interactive voice responses, we were able to collect only categorical data on travel duration. This, combined with the necessity of aggregating the six follow-up months, made it difficult to determine the total travel length. Sixth, entering a passcode to access the second HIV-related half of the survey proved challenging for many participants, and adding this extra layer of participant protection resulted in lower question completion rates and a smaller sample size for this subsection. Finally, it should be noted that Uganda’s model of hosting refugees offers refugees greater freedom of movement and livelihood pursuit than is granted by most other countries, which limits the generalizability of our findings.

## Conclusion

Refugees and Ugandan nationals accessing HIV care in refugee settlements in Uganda frequently traveled to meet their survival needs, and more than one in ten spent a prolonged period of time away from home in the past year. ART adherence may be challenging during travel, and mobility was associated with inferior retention in HIV care. Mobility should be considered in interventions to optimize HIV care in refugee settlements.

## Additional File

The additional file for this article can be found as follows:

10.5334/aogh.4367.s1 Appendices. Appendix 1 to 3. Appendix 1–Appendix 3.
