# Supplementary material for: Characterizing Mobility and its Association with HIV Outcomes in Refugee Settlements in Uganda
Source: Ann Glob Health. 2024 Mar 25;90(1):23. doi: 10.5334/aogh.4367 (PMC10976981; doi:10.5334/aogh.4367)
Supplement: Appendices. — Appendix 1 to 3. Appendix 1–Appendix 3. [file agh-90-1-4367-s1.pdf]

Appendix 1 Monthly mobility survey questions

Question 1	In the past 4 weeks, did the place where you live change?<br><br>If Yes, press 1.<br>If No, press 2.<br>If you do not know or prefer not to answer, press 0.
Question 2	In the past 4 weeks, did you spend any nights away from the place where you are living?<br><br>If Yes, press 1.<br>If No, press 2 → End of survey.<br>If you don't know or prefer not to answer, press 0.
Question 3	In the past 4 weeks, what was the total number of nights you spent away from the place where you are living?<br><br>If you spent 1 to 2 nights away, press 1.<br>If you spent 3 to 7 nights away, press 2.<br>If you spent more than 1 week to 2 weeks away, press 3.<br>If you spent more than 2 weeks to 3 weeks away, press 4.<br>If you spent more than 3 weeks away, press 5.<br>If you do not know or prefer not to answer, press 0.
Question 4	In the past 4 weeks, how many separate trips did you take? In other words, how many separate trips did you take where you spent at least one night away from the place where you live?<br><br>If you made one trip, press 1.<br>If you made two trips, press 2.<br>If you made three trips, press 3.<br>If you made four or more trips, press 4.<br>If you do not know or prefer not to answer, press 0.
Question 5	At this moment in time, are you on a trip spending at least one night away from the place where you live?<br><br>If yes, press 1.<br>If no, press 2.<br>If you do not know or prefer not to answer, press 0.
Question 6	What was the duration of your most recent trip or if you are currently traveling, how many nights have you been away so far?<br><br>If the duration was 1 to 2 nights, press 1.<br>If the duration was 3 to 7 nights, press 2.<br>If the duration was more than 1 week to 2 weeks, press 3.<br>If the duration was more than 2 weeks to 3 weeks, press 4.<br>If the duration was more than 3 weeks, press 5<br>If you do not know or prefer not to answer, press 0.
Question 7	What was the primary reason for your most recent travel?<br><br>If employment or trade, press 1.<br>If farming or looking for food, press 2.<br>If attending a function such as a wedding or a funeral, press 3.<br>If to be with partner or children or visiting extended family or friends, press 4.<br>If education or studies, press 5.<br>If seeking health care, press 6.<br>If touring, press 7.<br>If other, press 8.

	If you do not know or prefer not to answer, press 0.
Question 8	How far did you travel on your most recent trip?<br><br>If outside the village but within the parish, press 1.<br>If outside the parish but within the sub county, press 2.<br>If outside the sub county but within the district, press 3.<br>If outside the district, but within the region, press 4.<br>If outside the region, but within the country, press 5.<br>If outside of Uganda, press 6.<br>If you do not know or prefer not to answer, press 0.
Question 9	[if answered outside of Uganda]<br><br>Which country did you travel to?<br><br>If South Sudan, press 1.<br>If Sudan, press 2.<br>If the Democratic Republic of the Congo, press 3.<br>If Rwanda, press 4.<br>If Tanzania, press 5.<br>If Kenya, press 6.<br>If other, press 7.<br>If you do not know or prefer not to answer, press 0.
Question 10	How often do you take this trip?<br><br>If this is the first time, press 1.<br>If you take this trip at least once a week, press 2.<br>If you take this trip at least once a month, press 3.<br>If you take this trip at least every three months, press 4.<br>If you take this trip at least once every half year, press 5.<br>If you take this trip at least once a year, press 6.<br>If you take this trip less frequently than once a year, press 7.<br>If you do not know or prefer not to answer, press 0.
Question 11	On your most recent trip, were the people you were traveling with aware of your HIV status?<br><br>If Yes, press 1.<br>If No, press 2.<br>If you were traveling alone, press 3.<br>If you do not know or prefer not to answer, press 0.
Question 12	On your most recent trip, were the people you were staying with at your destination aware of your HIV status?<br><br>If Yes, press 1.<br>If No, press 2.<br>If you were staying alone, press 3.<br>If you do not know or prefer not to answer, press 0.
Question 13	Are you currently on anti-retroviral therapy, also known as ART for your HIV?<br><br>If you are taking ART, press 1.<br>If you started taking ART, but are not taking ART now, press 2.<br>If you are not on ART, press 3.<br>If you do not know or prefer not to answer, press 0.
Question 14	In preparation for you trip, did you receive more ART from HIV clinic than your usual amount?<br><br>If Yes, press 1.

	If No, press 2.<br>If you do not know or prefer not to answer, press 0.
Question 15	On your most recent trip, did you take your daily ART medication?<br><br>If you never took your ART medication on this trip, press 1.<br>If you sometimes took your ART medication on this trip, press 2.<br>If you most of the time took your ART medication on this trip, press 3.<br>If you always took your ART medication on this trip, press 4.<br>If you do not know or prefer not to answer, press 0.
Question 16	What was the main reason for not taking ART?<br><br>If you did not bring enough medication for the duration of the trip, press 1.<br>If your ART medication was lost or stolen, press 2.<br>If you forgot to take your ART medication, press 3.<br>If you were afraid others would become aware of your HIV status, press 4.<br>If you thought it was not necessary to take ART while traveling, press 5.<br>If you were busy with other activities, press 6.<br>If it was because of another reason, press 7.<br>If you do not know or prefer not to answer, press 0.
Question 17	On your most recent trip, did you miss more or fewer doses of ART than when you are at home?<br><br>If you missed more while traveling, press 1.<br>If you missed less while traveling, press 2.<br>If you missed the same while traveling, press 3.<br>If you do not know or prefer not to answer, press 0.
Question 18	Did you communicate in-person or by phone or text with anyone from the HIV clinic about your most recent trip before you departed?<br><br>If Yes, press 1.<br>If No, press 2.<br>If you do not know or prefer not to answer, press 0.
Question 19	Did you end up missing any HIV clinic appointments because of your most recent trip?<br><br>If Yes, press 1.<br>If No, press 2.<br>If you do not know or prefer not to answer, press 0.
Question 20	During your travels, did you seek care for HIV at a different health center than the one you normally go to?<br><br>If Yes, press 1.<br>If No, press 2.<br>If you do not know or prefer not to answer, press 0.

Appendix 2. Survey question completion

Question		Person-months (Total N=1,565) N (%)	Participants (Total N=479) N (%)
1	*In the past 4 weeks, did the place where you live change?*	1,552 (99.2)	479 (100)
2	*In the past 4 weeks, did you spend any nights away from the place where you are living?*<br><br>[if no → end survey]	1482 (94.7)	467 (97.5)
3	[If answered yes or do not know/prefer not to answer to Q2]<br>*In the past 4 weeks, what was the total number of nights you spent away from the place where you are living?*	530 (33.9)	297 (62.0)
4	[If answered yes or do not know/prefer not to answer to Q2]<br>*In the past 4 weeks, how many separate trips did you take? In other words, how many separate trips did you take where you spent at least one night away from the place where you live?*	513 (32.8)	289 (60.3)
5	[If answered yes or do not know/prefer not to answer to Q2]<br>*At this moment in time, are you on a trip spending at least one night away from the place where you live?*	488 (31.2)	273 (57.0)
6	[If answered yes or do not know/prefer not to answer to Q2]<br>*What was the duration of your most recent trip or if you are currently traveling, how many nights have you been away so far?*	476 (30.4)	268 (55.9)
7	[If answered yes or do not know/prefer not to answer to Q2]<br>*What was the primary reason for your most recent travel?*	468 (29.9)	262 (54.7)
8	[If answered yes or do not know/prefer not to answer to Q2]<br>*How far did you travel on your most recent trip?*	464 (29.6)	261 (54.5)
9	[If answered yes or do not know/prefer not to answer to Q2 AND answered outside of Uganda to Q8]<br>*Which country did you travel to?*	6 (<1)	5 (1.0)
10	[If answered yes or do not know/prefer not to answer to Q2]<br>*How often do you take this trip?*	459 (29.3)	258 (53.9)
11	[If answered yes or do not know/prefer not to answer to Q2]<br>*On your most recent trip, were the people you were traveling with aware of your HIV status?*	127 (8.1)	96 (20.0)
12	[If answered yes or do not know/prefer not to answer to Q2]<br>*On your most recent trip, were the people you were staying with at your destination aware of your HIV status?*	127 (8.1)	96 (20.0)
13	[If answered yes or do not know/prefer not to answer to Q2]<br>*Are you currently on anti-retroviral therapy, also known as ART for your HIV?*	126 (8.1)	96 (20.0)
14	[If answered yes or do not know/prefer not to answer to Q2]<br>*In preparation for you trip, did you receive more ART from HIV clinic than your usual amount?*	111 (7.1)	87 (31.2)
15	[If answered yes or do not know/prefer not to answer to Q2]<br>*On your most recent trip, did you take your daily ART medication?*	110 (7.0)	86 (18.0)
16	[If answered yes or do not know/prefer not to answer to Q2 AND did not answer always to Q15]<br>*What was the main reason for not taking ART?*	94 (6.0)	77 (16.1)
17	[If answered yes or do not know/prefer not to answer to Q2]<br>*On your most recent trip, did you miss more or fewer doses of ART than when you are at home?*	105 (6.7)	83 (17.3)
18	[If answered yes or do not know/prefer not to answer to Q2]<br>*Did you communicate in-person or by phone or text with anyone from the HIV clinic about your most recent trip before you departed?*	117 (7.5)	91 (19.0)
19	[If answered yes or do not know/prefer not to answer to Q2]<br>*Did you end up missing any HIV clinic appointments because of your most recent trip?*	116 (7.4)	90 (18.8)

20	[If answered yes or do not know/prefer not to answer to Q2]<br>*During your travels, did you seek care for HIV at a different health center than the one you normally go to?*	115 (7.3)	89 (18.6)

Appendix 3.1 Prospectively collected mobility survey responses by sex

	Female (N=1630)	Male (N=740)	P-value*
Home change in past 4 weeks			
No	646 (65.2%)	322 (72.5%)	<0.01
Yes	345 (34.8%)	122 (27.5%)	
Spent nights away from home past 4 weeks			
No	642 (65.6%)	297 (70.5%)	0.08
Yes	336 (34.4%)	124 (29.5%)	
Total # nights away from home in past 4 weeks			
1 to 2 nights away	198 (60.4%)	64 (48.9%)	0.23
3 to 7 nights away	79 (24.1%)	40 (30.5%)	
More than 1 week to 2 weeks away	25 (7.6%)	11 (8.4%)	
More than 2 weeks to 3 weeks away	11 (3.4%)	7 (5.3%)	
More than 3 weeks away	15 (4.6%)	9 (6.9%)	
# of trips in past 4 weeks			
1 trip	175 (54.7%)	69 (55.6%)	0.33
2 trips	83 (25.9%)	35 (28.2%)	
3 trips	44 (13.8%)	10 (8.1%)	
4 or more trips	18 (5.6%)	10 (8.1%)	
Currently on a trip			
No	142 (45.8%)	60 (48.4%)	0.70
Yes	168 (54.2%)	64 (51.6%)	
Duration of most recent trip			
1 to 2 nights	153 (50.7%)	51 (44.7%)	0.47
3 to 7 nights	116 (38.4%)	51 (44.7%)	
More than 1 week to 2 weeks	16 (5.3%)	3 (2.6%)	
More than 2 weeks to 3 weeks	7 (2.3%)	3 (2.6%)	
More than 3 weeks	10 (3.3%)	6 (5.3%)	
Primary reason for most recent travel			
Employment or trade	81 (25.5%)	48 (40.3%)	0.08
Farming or looking for food	149 (46.9%)	41 (34.5%)	
Attending a function (e.g. wedding or a funeral)	31 (9.7%)	7 (5.9%)	
To be with partner or children or visiting extended family or friends	18 (5.7%)	8 (6.7%)	
Education/studies	8 (2.5%)	5 (4.2%)	
Seeking healthcare	23 (7.2%)	6 (5.0%)	
Touring	2 (0.6%)	1 (0.8%)	
Other	6 (1.9%)	3 (2.5%)	
Distance of most recent travel			

	Female (N=1630)	Male (N=740)	P-value*
Outside the village but within the parish	109 (34.6%)	41 (36.0%)	0.87
Outside the parish but within the sub county	114 (36.2%)	37 (32.5%)	
Outside the sub county but within the district	34 (10.8%)	12 (10.5%)	
Outside the district, but within the region	34 (10.8%)	12 (10.5%)	
Outside the region, but within the country	13 (4.1%)	8 (7.0%)	
Outside of Uganda	11 (3.5%)	4 (3.5%)	
If outside the country, to which country			
South Sudan	0 (0%)	1 (33.3%)	0.20
Sudan	0 (0%)	1 (33.3%)	
DRC	1 (33.3%)	0 (0%)	
Rwanda	0 (0%)	1 (33.3%)	
Tanzania	2 (66.7%)	0 (0%)	
Trip frequency			
This is the first time	120 (39.5%)	45 (38.5%)	0.16
At least once a week	91 (29.9%)	27 (23.1%)	
At least once a month	41 (13.5%)	23 (19.7%)	
At least every three months	23 (7.6%)	9 (7.7%)	
At least once every half year	11 (3.6%)	9 (7.7%)	
At least once a year	7 (2.3%)	0 (0%)	
Less frequently than once a year	11 (3.6%)	4 (3.4%)	

*P-values based on Chi-squared test

Appendix 3.2 Prospectively collected mobility survey responses by food security status

	Not food insecure (N=1780)	Food insecure (N=615)	P-value*
Home change in past 4 weeks			
No	732 (67.8%)	244 (65.9%)	0.54
Yes	347 (32.2%)	126 (34.1%)	
Spent nights away from home past 4 weeks			
No	721 (68.7%)	225 (62.3%)	0.03
Yes	329 (31.3%)	136 (37.7%)	
Total # nights away from home in past 4 weeks			
1 to 2 nights away	179 (53.6%)	85 (65.9%)	0.09
3 to 7 nights away	96 (28.7%)	25 (19.4%)	
More than 1 week to 2 weeks away	30 (9.0%)	6 (4.7%)	
More than 2 weeks to 3 weeks away	12 (3.6%)	6 (4.7%)	
More than 3 weeks away	17 (5.1%)	7 (5.4%)	
# of trips in past 4 weeks			
1 trip	178 (54.9%)	68 (54.4%)	0.38
2 trips	92 (28.4%)	29 (23.2%)	
3 trips	37 (11.4%)	17 (13.6%)	
4 or more trips	17 (5.2%)	11 (8.8%)	
Currently on a trip			
No	148 (47.1%)	57 (45.6%)	0.85
Yes	166 (52.9%)	68 (54.4%)	
Duration of most recent trip			
1 to 2 nights	138 (46.8%)	68 (54.4%)	0.23
3 to 7 nights	125 (42.4%)	44 (35.2%)	
More than 1 week to 2 weeks	11 (3.7%)	8 (6.4%)	
More than 2 weeks to 3 weeks	9 (3.1%)	1 (0.8%)	
More than 3 weeks	12 (4.1%)	4 (3.2%)	
Primary reason for most recent travel			
Employment or trade	99 (31.8%)	31 (23.7%)	0.52
Farming or looking for food	127 (40.8%)	67 (51.1%)	
Attending a function (e.g. wedding or a funeral)	27 (8.7%)	11 (8.4%)	
To be with partner or children or visiting extended family or friends	20 (6.4%)	6 (4.6%)	
Education/studies	10 (3.2%)	3 (2.3%)	
Seeking healthcare	21 (6.8%)	8 (6.1%)	
Touring	2 (0.6%)	1 (0.8%)	
Other	5 (1.6%)	4 (3.1%)	

	Not food insecure (N=1780)	Food insecure (N=615)	P-value*
Distance of most recent travel			
Outside the village but within the parish	111 (36.3%)	40 (31.3%)	0.13
Outside the parish but within the sub county	108 (35.3%)	47 (36.7%)	
Outside the sub county but within the district	36 (11.8%)	10 (7.8%)	
Outside the district, but within the region	28 (9.2%)	18 (14.1%)	
Outside the region, but within the country	16 (5.2%)	5 (3.9%)	
Outside of Uganda	7 (2.3%)	8 (6.3%)	
If outside the country, to which country			
South Sudan	0 (0%)	1 (25.0%)	0.44
Sudan	1 (50.0%)	0 (0%)	
DRC	0 (0%)	1 (25.0%)	
Rwanda	0 (0%)	1 (25.0%)	
Tanzania	1 (50.0%)	1 (25.0%)	
Trip frequency			
This is the first time	122 (40.7%)	44 (34.9%)	0.39
At least once a week	80 (26.7%)	40 (31.7%)	
At least once a month	50 (16.7%)	15 (11.9%)	
At least every three months	22 (7.3%)	10 (7.9%)	
At least once every half year	13 (4.3%)	8 (6.3%)	
At least once a year	3 (1.0%)	4 (3.2%)	
Less frequently than once a year	10 (3.3%)	5 (4.0%)	

*P-values based on Chi-squared test

Appendix 3.3 Prospectively collected mobility survey responses by refugee/Ugandan national status

	Uganda national (N=1030)	Refugee (N=1365)	P-value*
Home change in past 4 weeks			
No	438 (74.1%)	538 (62.7%)	<0.001
Yes	153 (25.9%)	320 (37.3%)	
Spent nights away from home past 4 weeks			
No	422 (71.5%)	524 (63.8%)	<0.01
Yes	168 (28.5%)	297 (36.2%)	
Total # nights away from home in past 4 weeks			
1 to 2 nights away	92 (51.7%)	172 (60.4%)	0.18
3 to 7 nights away	49 (27.5%)	72 (25.3%)	
More than 1 week to 2 weeks away	19 (10.7%)	17 (6.0%)	
More than 2 weeks to 3 weeks away	6 (3.4%)	12 (4.2%)	
More than 3 weeks away	12 (6.7%)	12 (4.2%)	
# of trips in past 4 weeks			
1 trip	92 (51.7%)	154 (56.8%)	0.29
2 trips	50 (28.1%)	71 (26.2%)	
3 trips	27 (15.2%)	27 (10.0%)	
4 or more trips	9 (5.1%)	19 (7.0%)	
Currently on a trip			
No	73 (45.1%)	132 (47.7%)	0.67
Yes	89 (54.9%)	145 (52.3%)	
Duration of most recent trip			
1 to 2 nights	71 (45.8%)	135 (50.9%)	0.12
3 to 7 nights	61 (39.4%)	108 (40.8%)	
More than 1 week to 2 weeks	10 (6.5%)	9 (3.4%)	
More than 2 weeks to 3 weeks	7 (4.5%)	3 (1.1%)	
More than 3 weeks	6 (3.9%)	10 (3.8%)	
Primary reason for most recent travel			
Employment or trade	48 (30.0%)	82 (29.1%)	0.15
Farming or looking for food	61 (38.1%)	133 (47.2%)	
Attending a function (e.g. wedding or a funeral)	18 (11.3%)	20 (7.1%)	
To be with partner or children or visiting extended family or friends	11 (6.9%)	15 (5.3%)	
Education/studies	5 (3.1%)	8 (2.8%)	
Seeking healthcare	12 (7.5%)	17 (6.0%)	
Touring	3 (1.9%)	0 (0%)	
Other	2 (1.3%)	7 (2.5%)	

	Uganda national (N=1030)	Refugee (N=1365)	P-value*
Distance of most recent travel			
Outside the village but within the parish	57 (35.4%)	94 (34.4%)	0.15
Outside the parish but within the sub county	50 (31.1%)	104 (38.5%)	
Outside the sub county but within the district	22 (13.7%)	24 (8.8%)	
Outside the district, but within the region	22 (13.7%)	24 (8.8%)	
Outside the region, but within the country	7 (4.3%)	14 (5.1%)	
Outside of Uganda	3 (1.9%)	12 (4.4%)	
If outside the country, to which country			
South Sudan	0 (0%)	1 (20.0%)	0.20
Sudan	1 (100%)	0 (0%)	
DRC	0 (0%)	1 (20.0%)	
Rwanda	0 (0%)	1 (20.0%)	
Tanzania	0 (0%)	2 (40.0%)	
Trip frequency			
This is the first time	53 (33.5%)	113 (42.3%)	0.08
At least once a week	45 (28.5%)	75 (28.0%)	
At least once a month	31 (19.6%)	34 (12.7%)	
At least every three months	15 (9.5%)	17 (6.3%)	
At least once every half year	10 (6.3%)	11 (4.1%)	
At least once a year	2 (1.3%)	5 (1.9%)	
Less frequently than once a year	2 (1.3%)	13 (4.9%)	

*P-values based on Chi-squared test
